# Cardiac computer tomography-derived radiomics in assessing myocardial characteristics at the connection between the left atrial appendage and the left atrium in atrial fibrillation patients

**DOI:** 10.3389/fcvm.2024.1442155

**Published:** 2025-01-13

**Authors:** Xiao-Xuan Wei, Cai-Ying Li, Hai-Qing Yang, Peng Song, Bai-Lin Wu, Fang-Hua Zhu, Jing Hu, Xiao-Yu Xu, Xin Tian

**Affiliations:** ^1^ Department of Medical Imaging, The Second Hospital of Hebei Medical University, Shijiazhuang, China; ^2^ Department of Statistical Investigation, Statistical Information Center of Hebei Health Commission, Shijiazhuang, China

**Keywords:** atrial fibrillation (AF), radiomics, myocardial thickness, cardiac CT, left atrial appendage (LAA)

## Abstract

**Objectives:**

To evaluate the feasibility of utilizing cardiac computer tomography (CT) images for extracting the radiomic features of the myocardium at the junction between the left atrial appendage (LAA) and the left atrium (LA) in patients with atrial fibrillation (AF) and to evaluate its asscociation with the risk of AF.

**Methods:**

A retrospective analysis was conducted on 82 cases of AF and 56 cases in the control group who underwent cardiac CT at our hospital from May 2022 to May 2023, with recorded clinical information. The morphological parameters of the LAA were measured. A radiomics model, a clincal feature model and a model combining radiomics and clinical features were constructed. The radiomics model was built by extracting radiomic features of the myocardial tissue using Pyradiomics, and employing Least absolute shrinkage and selection operator (LASSO) method for feature selection, combining random forest with support vector machine (SVM) classifier.

**Results:**

There were 82 cases in the AF group [44 males, 65.00 (59, 70)], and 56 cases in the control group (21 males, 61.09 ± 7.18). Age, BMI, hypertension, CHA2DS-VASC score, neutrophil to lymphocyte ratio (NLR), LAA volume, LA volume, the myocardial thickness at the junction of LAA and LA, the area, circumference, short diameter, and long diameter of the LAA opening, were significantly different between the AF group and the control group (*P* < 0.05). After conducting multivariate logistic regression analysis, it was found that BMI, the myocardial thickness at the junction of the LAA and the LA, LA volume, NLR and CHA2DS-VASC score were related to AF. 12 radiomics features of the myocardium at the junction of the LAA and the LA were extracted and identified. ROC curve analysis confirmed that the nomogram based on radiomics scores and clinical factors can effectively predict AF (AUC 0.869).

**Conclusion:**

Radiomics enables the extraction of the myocardial characteristics at the junction of the LAA and the LA, which are related with AF, facilitating the assessment of its relationship with the risk of AF. The combination of radiomics with clinical characteristics enhances the evaluation capabilities significantly.

## 1 Introduction

AF is one of the most common sustained arrhythmias, with a prevalence of approximately 1%–2% (1). In severe cases, it can cause complications such as thromboembolism and heart failure in patients (2, 3). Therefore, early identification of AF for intervention and treatment is of great significance. Recent research has elucidated the significance of the morphology and structural features of the LA and LAA in the onset and progression of AF (4).

The LAA is a residual irregular and highly trabecular tissue of the embryonic LA that develops in early embryonic development. It is connected to the LA through a narrow foramen (5, 6). The persistent stimulation of AF prompts various myocardial cells to release multiple factors, leading to cardiomyocyte hypertrophy and fibrosis (7), which contribute to myocardial remodeling. Previous studies have shown that LA myocardium is involved in the pathological remodeling progress (8). Therefore, it is believable that the myocardium situated at the junction of the LAA and LA is remodel because of fibrosis. Consequently, assessing the myocardial characteristics at the junction holds promise for predicting the risk of AF.

Radiomics is defined as extracting numerous image features based on computer tomography (CT), and magnetic resonance imaging (MRI) after converting image information into data information (9, 10). Then the radiomics features is screened out by statistical software and other methods to build radiomics models for diagnosis and prognosis assessment (11), including distinguishing between benign and malignant tumors, lymph node metastasis, and prognosis analysis (12–14). Least absolute shrinkage and selection operator (LASSO) is a regression technique for variable selection and regularization to enhance the prediction accuracy. LASSO regression adds a penalty equal to the absolute value of the magnitude of coefficients, and some coefficients can become zero and are eventually eliminated from the model, resulting in variable elimination, and thus models with fewer coefficients, to feature screening (15, 16). After feature selection, it will obtain radiomics features that have a significant impact on diagnosis, prognosis, and other results (17).

Radiomics models have been used to analyze epicardial tissue, including adipose and myocardium. Previous studies have proposed radiomics models based on CTA and CMR to predict AF and AF recurrence. A radiomics model based on CT was used to analyze features of epicardial adipose tissue surrounding the left atrium (LA-EAT). Combined with the volume information of LA, it can effectively distinguish subtypes of AF and predict recurrence (18). CMR can also be used to extract radiomics features of the volume and surface of the LA in the end diastole phase. The radiomics model, combined with ECG parameters, can predict the risk of AF, particularly in female patients (19). The recurrence of AF after ablation can be predicted based on the fractal features of the LA and pulmonary veins, as well as the LA wall (20). However, radiomic analysis focused on myocardial thickness at the junction of the left atrial appendage (LAA) and left atrium (LA) has not yet been explored. Our approach introduces this novel indicator, providing a new avenue for AF prediction. The aim of this study is to extract the radiomics characteristics of the myocardium at the junction between the LAA and the LA in patients with AF, and combine radiomics with the clinical characteristics to predict the risk of AF.

## 2 Materials and methods

### 2.1 Subjects

A retrospective analysis was conducted on 82 cases of AF and 56 cases of controls who underwent cardiac CT scans at the Second Hospital of Hebei Medical University between May 2022 and May 2023. The study complied with the Declaration of Helsinki and was approved by the Institutional Ethics Committee of the Second Hospital of Hebei Medical University.

Inclusion criteria for patients in the AF group: (1) All patients were clinically diagnosed with AF by physical examination and electrocardiogram; (2) All patients had undergone 256-slice cardiac CT examination. Exclusion criteria for patients in the AF group: (1) Poor cardiac CT image quality with unclear myocardium visualization. (2) Contraindications for cardiac CT, including patients with cardiac implants. (3) Presence of valvular heart disease, congenital heart disease, cardiomyopathy, or myocarditis. Inclusion criteria for the control group: (1) No evidence of cardiomyopathy. (2) cardiac CT images indicating the absence of coronary artery sclerosis. Exclusion criteria: (1) Poor cardiac CT image quality. (2) Allergy to iodine contrast media or presence of pacemakers/other internal devices. (3) History of myocardial infarction.

### 2.2 Clinical data

Patient clinical data were recorded, including age, gender, Body mass index (BMI), CHA2DS-VASC score (21) (Table 1) (congestive heart failure, hypertension, age ≥ 75 years, diabetes, Previous stroke, transient ischemic attack, or thromboembolism), NLR (neutrophil/lymphocyte), hypertension and diabetes (22).

**Table 1:** CHA2DS-VASC score.

Risk factors	Score
Congestive Heart Failure/Left ventricular dysfunction	1
Hypertension	1
Age ≥75 years	2
Diabetes mellitus	1
Stroke	2
Vascular disease	1
Age 65–74 years	1
Sex (female sex)	1

### 2.3 Cardiac CT acquisition

Scanning was performed using Philips 256-slice spiral CT, with an image resolution of 5.12 Lp/cm, The slice thickness was 0.9 mm, the slice spacing was 0.45 mm. During CT scan, patients were positioned supine, employing retrospective ECG gating technology and a single end-expiration breath-hold scan to accurately control the scanning range. The non-ionic contrast agent iohexol (350 mgl/ml, dose 0.8 ml/kg) was injected intravenously into median cubital vein, and the scanning range is 0.5 cm below the tracheal bifurcation to the diaphragm. Scanning parameters: tube voltage 80∼120 kV, tube current 280∼350 mAs/revolution, collimation 128 × 0.625, pitch 0.18, rotation time 330 ms, matrix 512 × 512, field of view 250 mm.

### 2.4 Image post-processing and measurement

A comprehensive cardiac analysis (CCA) cardiac function processing software on the Philips EBW 4.5 workstation was used for image post-processing and measurement. The original images of 75% phase of the cardiac cycle were used to identify the LA and LAA, obtaining three-dimensional images of the LA and LAA. The myocardial thickness at the junction of the LAA and the LA, the volume of the LAA and the LA, and the LAA depth were measured. The LAA base opening was observed using multi-plane reconstruction technology (MPR).

**Figure 1:**
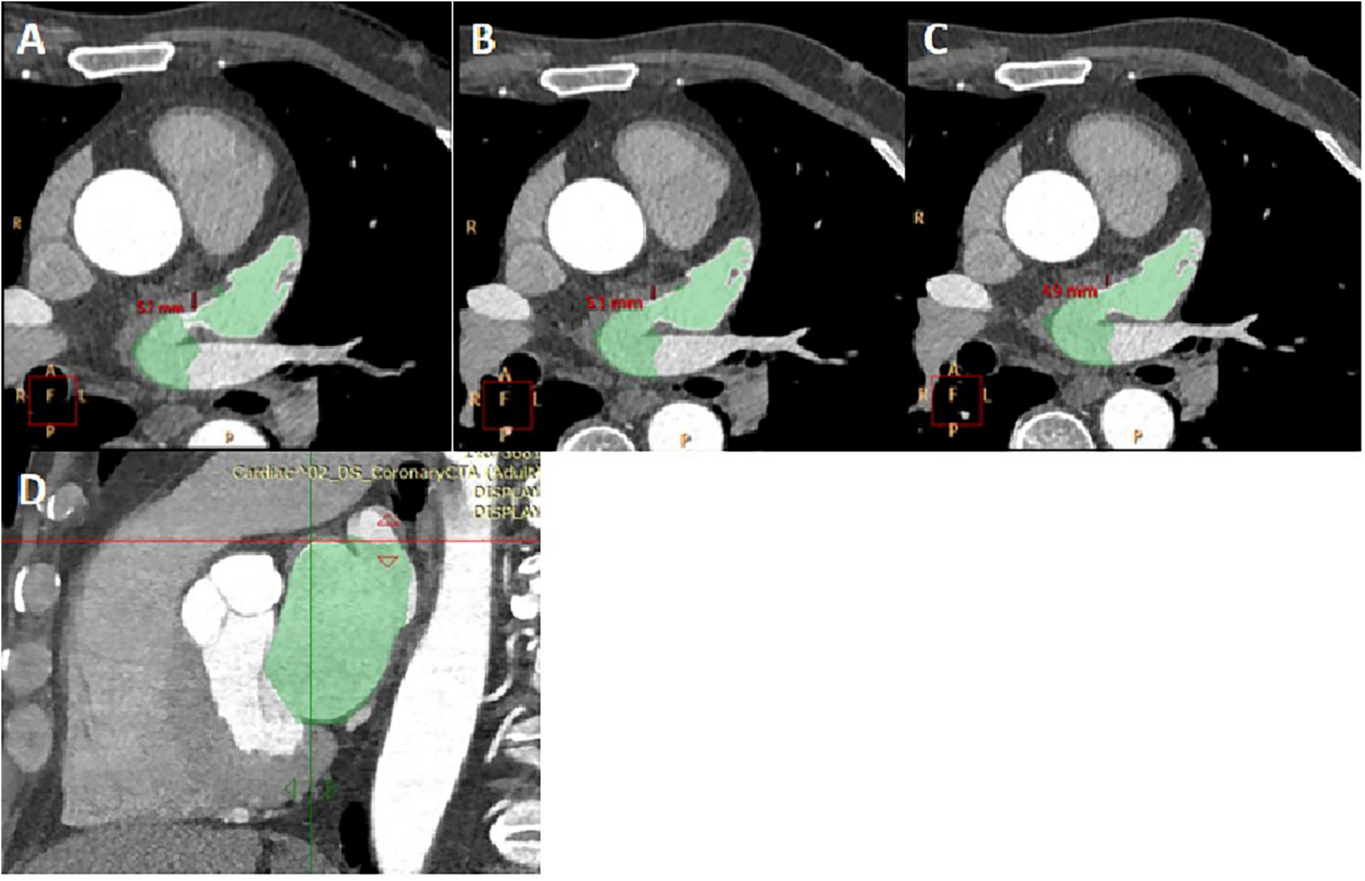
The measurement of myocardial thickness at the junction of the LAA and the LA. **(A–C)** three consecutive cross-sectional images of myocardium at the junction of the LAA and the LA was shown. The thickness of myocardium on three layers was measured, and Figure 1A was determined to be the thickest myocardium layer. LA and LAA are colored. **(D)** A sagittal image displayed the thickest part of the myocardium, indicated by the crossroads. It was located by positioning the marker on the myocardium of the aforementioned cross-section.

Measurement of LA and LAA parameters: Three planes of the heart were obtained on the Philips EBW 4.5 workstation. Initially, the thickness of the myocardium at the junction of the LAA and the LA was measured on multiple consecutive cross-sectional plane images (Figures 1A–C). The thickest layer of the myocardium was identified at the isthmus of the LAA. (Figure 1A) (23). Then, by positioning the marker on the thickest myocardium on the cross-sectional plane image, the marker automatically placed on the corresponding location on the sagittal image (Figure 1D). Finally, it was verified on the sagittal image to ensure the thickest layer of the myocardium.

**Figure 2:**
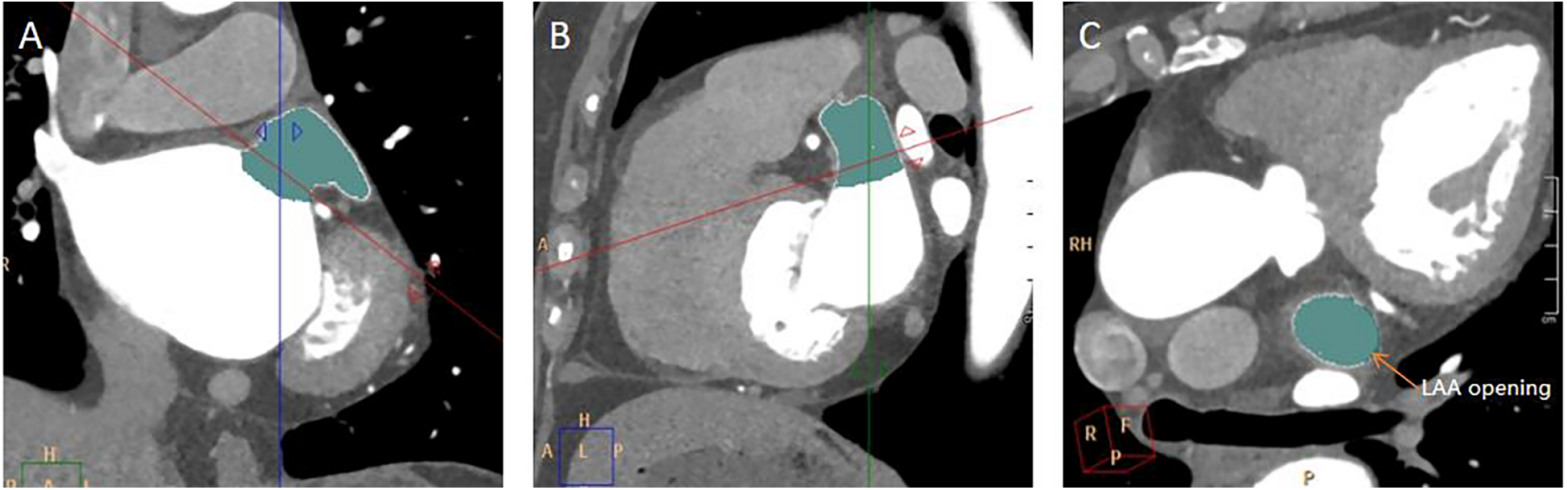
Measurement of LAA opening. **(A)** A coronal image of the LAA opening. The red line was along the plane of the LAA opening. **(B)** A sagittal image of the LAA opening. **(C)** A transverse image of the LAA opening.

**Figure 3:**
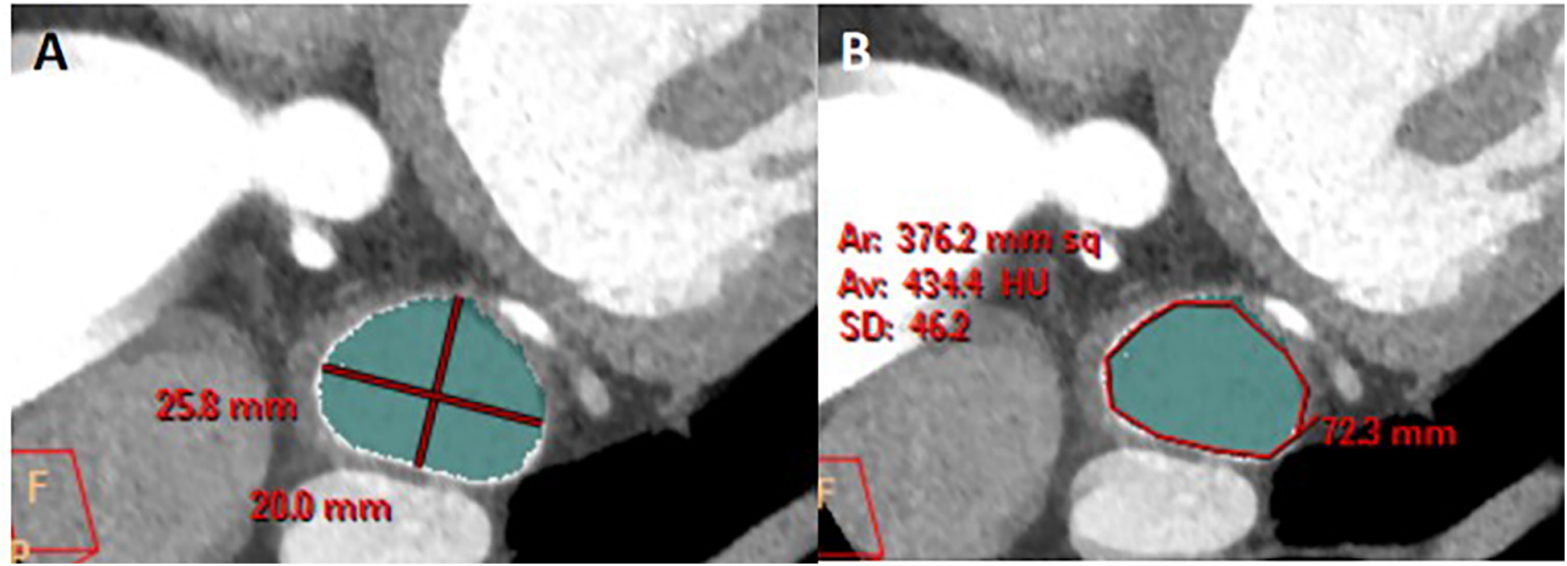
Measurement of the long diameter, short diameter, circumference, and area of the LAA opening. **(A)** On the plane depicting the LAA opening in Figure 2C, the longest diameter of the LAA opening was measured, and a line segment perpendicular to it was drawn to represent the short diameter of the LAA opening. **(B)** On the same image of A, the circumference and area of the LAA opening was automatically calculated by outlining the opening of the LAA.

The long diameter, short diameter, area, and circumference of the opening of the LAA were measured. Find the maximum plane at the junction of the LAA and the LA in the coronal plane, perpendicular the positioning line to the junction of the LAA and the LA (Figure 2A). Then, on the sagittal plane image, perpendicular the positioning line to the junction of the LAA and the LA (Figure 2B). Utilizing the MPR technique and finally a cross-sectional image of the LAA opening was obtained (Figure 2C). The long diameter, short diameter, area, and circumference of the LAA base opening were measured on this cross-sectional image of the LAA opening (Figure 3).

**Figure 4:**
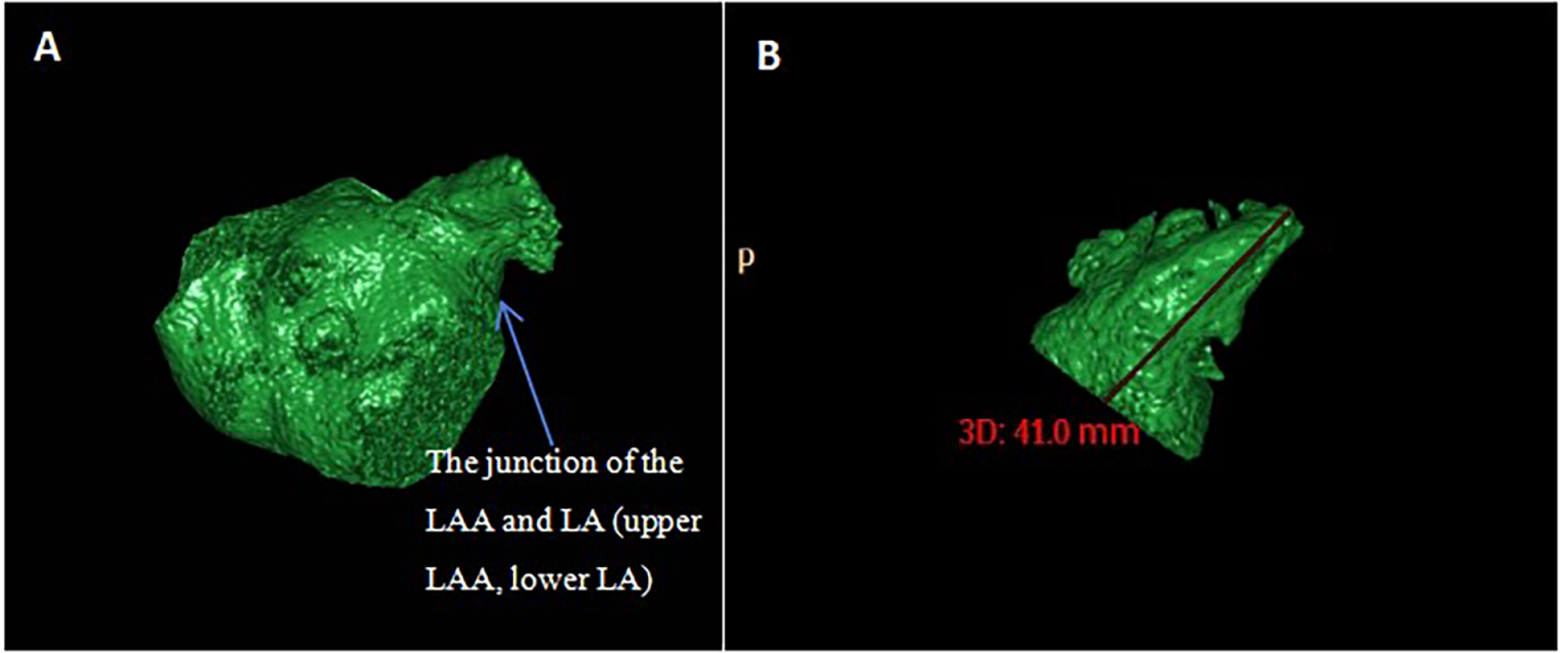
Measurement of LAA volume, LA volume, and LAA depth. **(A)** The post-processing software automatically calculate the volume of the LAA and the LA. LAA was segmented at the connection between the LAA and the LA. **(B)** LAA was obtained separately. The length from the tip of the LAA to the midpoint of the LAA opening was measured as the depth of the LAA.

Measurement of LAA and LA volumes. The CCA software automatically calculates the total volume of the LA and LAA (Figure 4A). Subsequently, the LAA was isolated by segmenting it at the root. The root of the LAA refers to the narrow region or base connecting the LAA to the main body of the LA. The root of the LAA was defined as the site of reflection of this structure with the surrounding LA wall (24). After that, the volume of the LAA was obtained automatically (Figure 4B), and the volume of the LA was calculated by subtracting the volume of the LAA from the total volume.

Measurement of the LAA depth diameter. The distance from the farthest point of the LAA tip to the center of the LAA opening plane was measured on a separate 3D image of the LAA (Figure 4B).

### 2.5 Radiomics feature extraction

**Figure 5:**
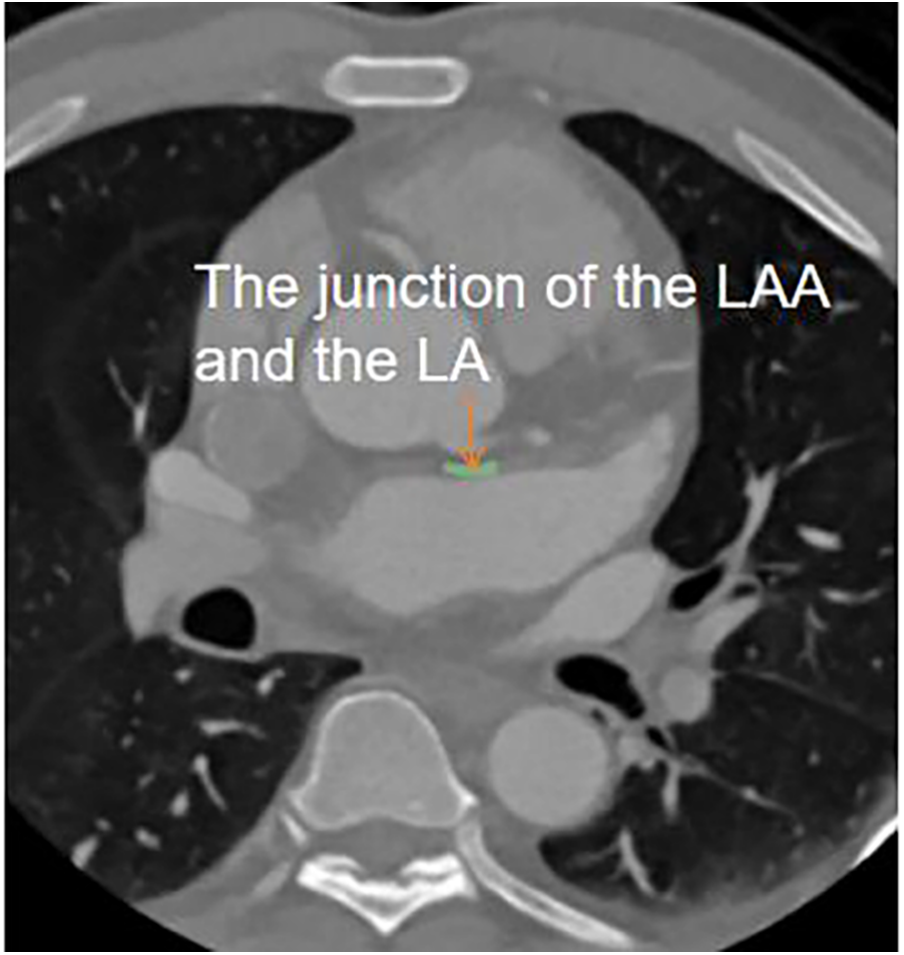
ROI delineation. On the axial image of cardiac CT, the myocardium at the junction of the LAA and the LA is delineated (green). The length of ROI was 1 cm in total.

In this study, the 3D-slicer software was utilized to delineate region of interest (ROI) on five or six consecutive axial images, which optimally visualized the myocardium at the junction of the LAA and LA. The center of ROI was designated as the junction point of the LAA and LA. Subsequently, the ROI was extended 1 cm along both sides of the LAA and LA, and the myocardium within this specified range was outlined (Figure 5).

Following ROI delineation, the Python software, specifically the Pyradiomics package, was employed to extract radiomics features and generate a dataset. For each patient, 112 radiomics features were extracted, encompassing first-order features, shape features, Gray Level Co-occurrence Matrix (GLCM) features, Gray Level Size Zone Matrix (GLSZM) features, Gray Level Run Length Matrix (GLRLM) features, and neighboring gray level dependence matrix (NGLDM).

### 2.6 Radiomics feature selection and model establishment

LASSO is used to select the most predictive radiomics features by reducing the regression coefficients of certain variables to zero through regularization penalties, thereby eliminating less important features (16, 25, 26). The PyRadiomics toolkit facilitates feature extraction and selection. Start by normalizing the data and then randomly split it into training and testing sets in a 7:3 ratio. Use the *T*-test and LASSO algorithms within the toolkit to perform dimensionality reduction on the normalized radiomics training data. From this analysis, select the radiomics feature with the highest predictive value, construct a radiomics signature based on the final selected feature, and generate radiomics scores (Rad scores) for each patient by weighting the selected feature values by their corresponding non-zero coefficients. Next, construct a radiomics feature model using random forest and support vector machine (SVM) classifiers. 10-fold cross-validation was performed on the training set (97 samples) to find the most appropriate parameters.

$$J(w)=\frac{1}{m}\sum_{i=1}^{m}{\left(y_{i}-w^{T}x_{i}\right)}^{2}+\lambda \sum_{i=1}^{m}\left|w_{i}\right|$$

*note: Cost Function. Yi, prediction result; Xi, corresponds to each eigenvalue of Yi; Wi, corresponds to the coefficient of each eigenvalue.

Calculate radiomics score:

Radiomics Score = β0 + β1X1 + β2X2 + β3X3 + .. + βnXn
*note: In the above formula, Xn represents the radiomics feature value with the most predictive value screened out in the LASSO regression model, and βn is the corresponding weighting coefficient of the corresponding radiomics feature in this regression model. The radiomics score of each patient can be calculated based on this formula.

## 3 Statistical analysis

All data were statistically processed using SPSS 21.0 statistical software, with econometric data presented as mean ± standard deviation for normality and median (interquartile range) for non-normality. Count data were described statistically using composition ratios. Statistical comparisons were conducted between the measurement data of the AF group and the control group. The measurement data underwent two independent sample *t*-tests for normality and homogeneity of variance, while the Mann-Whitney U rank sum test was used for non-normality or heterogeneity of variance; count data underwent chi-square tests. For confounding factors, a multiple factor logistic regression analysis was conducted to determine their degree of influence, with a *P* < 0.05 indicating a statistically significant difference. Separate correlation analyses were conducted between LA volume, onset time of AF, and the myocardial thickness at the junction of LAA and LA. Univariate logistic regression analysis was carried out on the statistical clinical data, selecting feature variables with *P* < 0.05, and establishing a multivariate logistic regression model. A nomogram was employed to evaluate the combination of radiomics and clinical features model for the risk of AF. The performance of the nomogram was assessed by calibrating the curve and the area under the ROC curve (AUC). Finally, a decision curve was used to evaluate the clinical benefit of the model. R Studio program was utilized to draw nomograms, calibration curves, and decision curves.

## 4 Results

### 4.1 Comparison of clinical data between the AF group and control group

**Figure 6:**
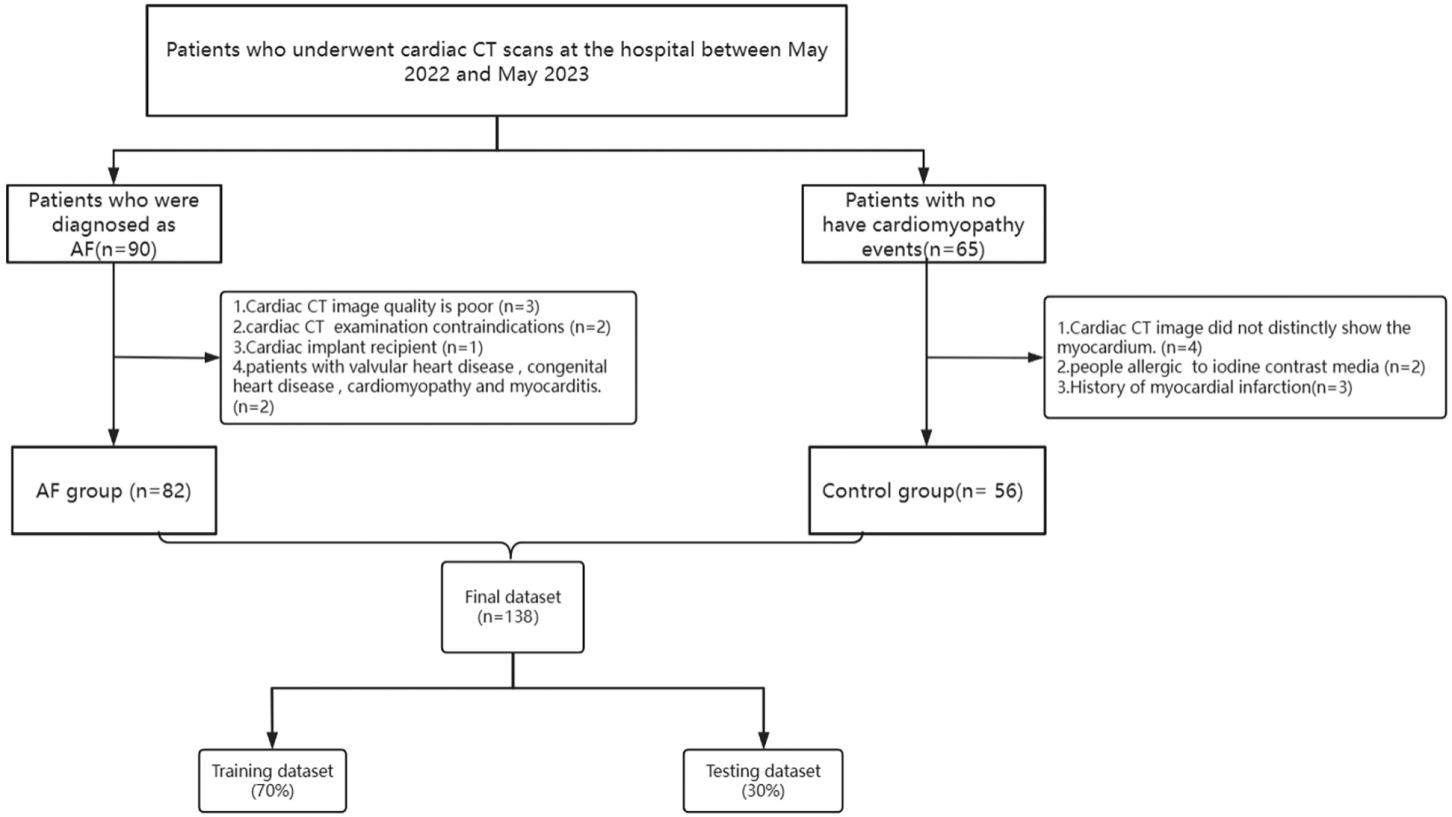
Flowchart of patient recruitment and study design. AF, Atrial fibrillation.

This study initially involved 90 patients with AF and 65 patients in the control group. Due to poor image quality and other reasons, 8 or 9 patients were excluded from each group, respectively. Consequently, a total of 82 patients were enrolled in the AF group and 56 patients were enrolled in the control group (Figure 6).

The clinical data of patients with AF and control group were compared. The patients with AF were older than the control group (Z/T = −3.03, *P* = 0.004). There were more cases of hypertension in the AF group than the control group (Z/T = 9.82, *P* = 0.002). BMI was slightly higher (Z/T = 3.21, *P* = 0.002) and NLR (Z/T = −4.79, *P* < 0.001) was lower in the AF group. The CHA2DS-VASC score (Z/T = −5.51, *P* < 0.001) was much higher in the AF group.In this study, patients with AF were older, had a high prevalence of hypertension, larger BMI, higher CHA2DS-VASC score and lower NLR. In the meanwhile, there was no statistical difference in gender and diabetes between the AF group and the control group (*P* ≥ 0.05) (Table 2).

**Table 2:** Comparison of clinical data between the AF group and the control group.

Variable	AF group	Control group	Z/T	*P*
Age (years)*	65.00 (59.0, 70.0)	61.09 ± 7.18	−3.03	0.004
Male, *n* (%)	44 (53.7%)	21 (37.5%)	3.82	0.51
Hypertension, *n* (%)*	59 (72.0%)	25 (44.6%)	9.82	0.002
Diabetes, *n*(%)	25 (30.4%)	12 (21.1%)	0.286	0.593
BMI*	26.98 ± 3.51	25.11 ± 3.09	3.21	0.002
Time of onset of AF	359 (333, 439.3)	/	/	/
CHA2DS-VASC score*	4.00 (2.00, 5.00)	1.36 (1.00, 3.00)	−5.51	*P* < 0.001
NLR*	1.49 (1.37, 1.62)	2.61 (1.43, 3.62)	−4.79	*P* < 0.001

* *P* < 0.05.

### 4.2 Comparison of LAA morphological parameters and LA volume between the AF group and the control group

The results showed that the AF group had a larger LAA opening area (*P* = 0.001), circumference (*P* < 0.001) than the control group and the short diameter (*P* < 0.001), long diameter (*P* = 0.002) are also longer. The LAA volume (*P* = 0.001), LA volume (*P* < 0.001) are also larger, and the myocardial thickness at the junction of LAA and LA(*P* < 0.001) was thicker than the control group.The LAA opening area, circumference, long and short diameter, myocardial thickness, the LAA and LA volume in patients with AF were all larger than those in the control group. The LAA was prone to involvement when AF occurred. There was no significant difference in LAA depth between the two groups (*P* > 0.05) (Table 3).

**Table 3:** Comparison of LAA morphological parameters and LA volume between the AF group and the control group.

Variable	AF group	Control group	*P*
LAA opening area (mm^2^)*	354.40 (260.35, 462.15)	291.60 ± 91.93	0.001
LAA opening circumference (mm)*	70.63 ± 13.95	63.30 ± 9.43	*P* < 0.001
LAA opening short diameter (mm)*	18.90 (15.25, 21.95)	16.11 ± 3.20	*P* < 0.001
LAA opening long diameter (mm)*	26.29 ± 5.01	23.89 ± 3.52	0.002
LAA depth (mm)	38.5 (35.18, 42.43)	37.13 ± 4.69	0.79
myocardial thickness (mm)*	6.85 (6.00, 8.53)	5.93 ± 1.37	*P* < 0.001
LAA volume (mm^3^)*	7.45 (5.57, 10.00)	5.90 (4.70, 7.60)	0.001
LA volume (mm^3^)*	102.30 (75.35, 131.60)	71.50 ± 14.15	*P* < 0.001

* *P* < 0.05.

### 4.3 Multivariate logistic regression of parameters between the AF group and the control group

In Table 4, B is the Beta coefficient, indicating the direction and magnitude of the relationship between each predictor and the outcome. A positive B value represents a positive correlation, while a negative B value represents a negative correlation. Odds Ratio (OR) quantifies the strength and direction of the association between each predictor and the likelihood of the AF. Multivariate analysis revealed that BMI (*P* = 0.046), the myocardial thickness at the junction of LAA and LA (*P* = 0.017), LA volume (*P* = 0.004), NLR (*P* = 0.001), and CHA2DS-VASC score (*P* = 0.029) demonstrated statistically predictors of AF.

**Table 4:** Multivariate logistic regression analysis of different parameters in the AF group and the control group.

	B	OR	*P*
Age	−0.062	0.940	0.266
Hypertension	−0.150	0.860	0.839
BMI*	0.223	1.250	0.046
LAA opening area	−0.006	0.994	0.734
LAA opening circumference	−0.308	0.735	0.052
LAA opening short diameter	0.486	1.627	0.102
LAA opening long diameter	0.437	1.549	0.125
Myocardial thickness*	0.653	1.922	0.017
LAA volume	0.309	1.362	0.152
LA volume*	0.059	1.060	0.004
CHA2DS-VASC score*	0.816	2.261	0.029
NLR*	−2.533	0.079	0.001

* *P* < 0.05.

BMI: Each 1 kg/m^2^ increase was associated with a 1.250-fold increase in the likelihood of AF. Myocardial Thickness: Every 1 mm increase at the junction of the LAA and LA increased AF risk by 1.922 times. LA Volume: Each 1 mm^3^ increase was linked to a 1.060-fold higher risk of AF. NLR: Every 1-unit increase in NLR was associated with a 0.079-fold decrease in AF risk. CHA2DS2-VASc Score: Each additional point was linked to a 2.261-fold increase in AF risk (Table 4).

### 4.4 Comparison of Rad-score between the AF group and the control group

The results showed that the AF group had a low score (*P* < 0.001) (Table 5).

**Table 5:** Comparison of Rad-score between the AF group and the control group.

	AF group	Control group	Z	*P*
Rad-score	75.33 (53.14, 98.70)	130.3964 ± 5.47	−5.901	*P* < 0.001

### 4.5 Correlation analysis between LA volume, onset time of AF and the myocardial thickness at the junction of LAA and LA

There was a significant positive correlation between LA volume and the myocardial thickness at the junction of LAA and LA (*P* = 0.001). The myocardial thickness did not correlated with AF onset time (*P* > 0.05) (Table 6).

**Table 6:** Correlation between LA volume, onset time of AF and myocardial thickness at the junction of LA and LAA.

Variable	LA volume*	*P*	AF onset time	*P*
Myocardial thickness	0.324	0.0001	0.079	0.356

* *P* < 0.05.

* AF onset time was defined as follows: if the patient did not receive radiofrequency ablation treatment, the AF onset time was calculated from the diagnosis time to the cardiac CT time; if the patient received radiofrequency ablation treatment, the AF onset time was calculated from the diagnosis time to the treatment time point.

### 4.6 Radiomics feature extraction of the myocardium in patients with AF

**Figure 7:**
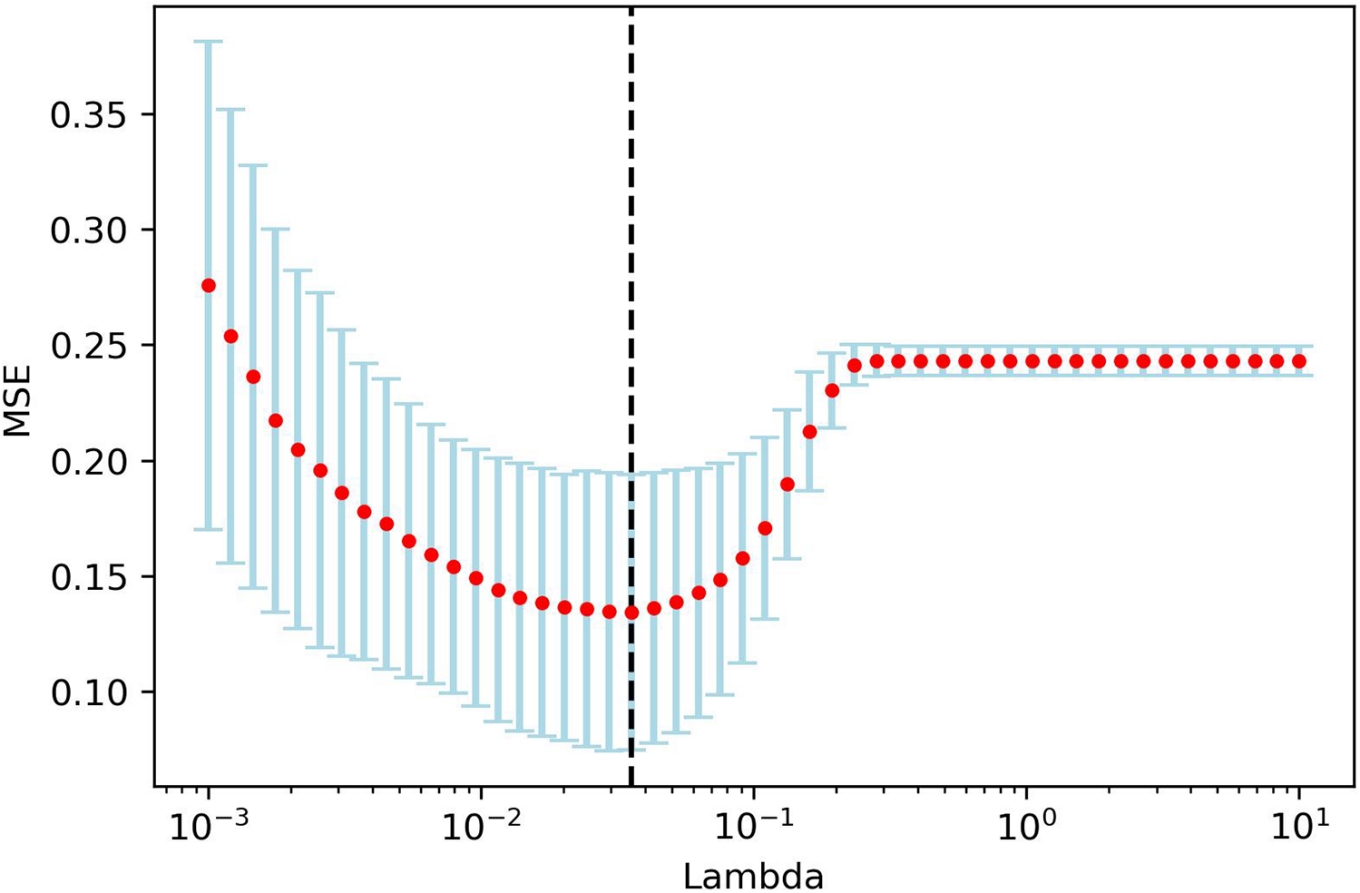
LAASO regression model dimensionality reduction. Lambda(*λ*) in the shrinkage coefficient represented the adjustment parameter. The vertical axis represented the mean square error, and the horizontal axis represents *λ*.

One hundred and twelve candidate radiomic features were extracted of each patient. After the features were normalized, the LASSO regression was used for feature selecting. Through LASSO regression, suitable variables were selected from 112 radiomics features, and the prediction effect was best when *λ* reached the minimum. A total of 14 variables (Table 7) were selected from all of the radiomics features Figure 7.

**Table 7:** Variable name and weight value.

Variable	Feature	Weight
original_shape_Maximum2DDiameterRow	feature1	−0.005
original_shape_Maximum3DDiameter	feature2	−0.022
original_shape_MinorAxisLength	feature3	−0.158
original_shape_SurfaceVolumeRatio	feature4	0.024
original_firstorder_Kurtosis	feature5	−0.01
original_glcm_Autocorrelation	feature6	0.023
original_glcm_Imc2	feature7	−0.083
original_glcm_JointEntropy	feature8	0.027
original_glcm_MaximumProbability	feature9	−0.027
original_glrlm_RunLengthNonUniformity	feature10	0.138
original_glszm_ZoneEntropy	feature11	0.011
original_ngtdm_Complexity	feature12	0.001
Unname	feature13	−0.014
diagnostics-Image-original_Mean	feature14	0.019

Some values obtained from the PyRadiomics library are redundant. lt is important to eliminate such redundant features prior to the machine learning and feature selection processes. As a result, two of these redundant parameters are among the 14 important features identified by their predictive model and regularization term. One is Unname, and the other is diagnostics-Image-original_Mean. They represented radiomics features of raw data, instead of myocardial features.

## 5 Building predictive models

Three predictive models were established to predict AF, including a model driven by clinical features, a radiomics model and a combined model integrating radiomics with clinical features.

### 5.1 The predictive model based on clinical data

Supported by univariate and multivariate logistic regression analysis, BMI, LA volume, the myocardial thickness at the junction of LAA and LA, NLR, and CHA2DS-VASC score were found to be associated with the occurrence of AF. R studio software was used to establish a clinical multivariate logistic regression model (As shown in the following code).

lrm(group∼ NLR + BMI + LAV + thickness + C, data = training_dataset, *x* = TRUE, *y* = TRUE,maxit = 1,000)
*note: lrm() is used to construct the logistic model function, Constructing a logistic regression model by incorporating risk factors (NLR, BMI, LAV, thickness, C) that have an impact on AF.

### 5.2 Radiomics score

**Figure 8:**
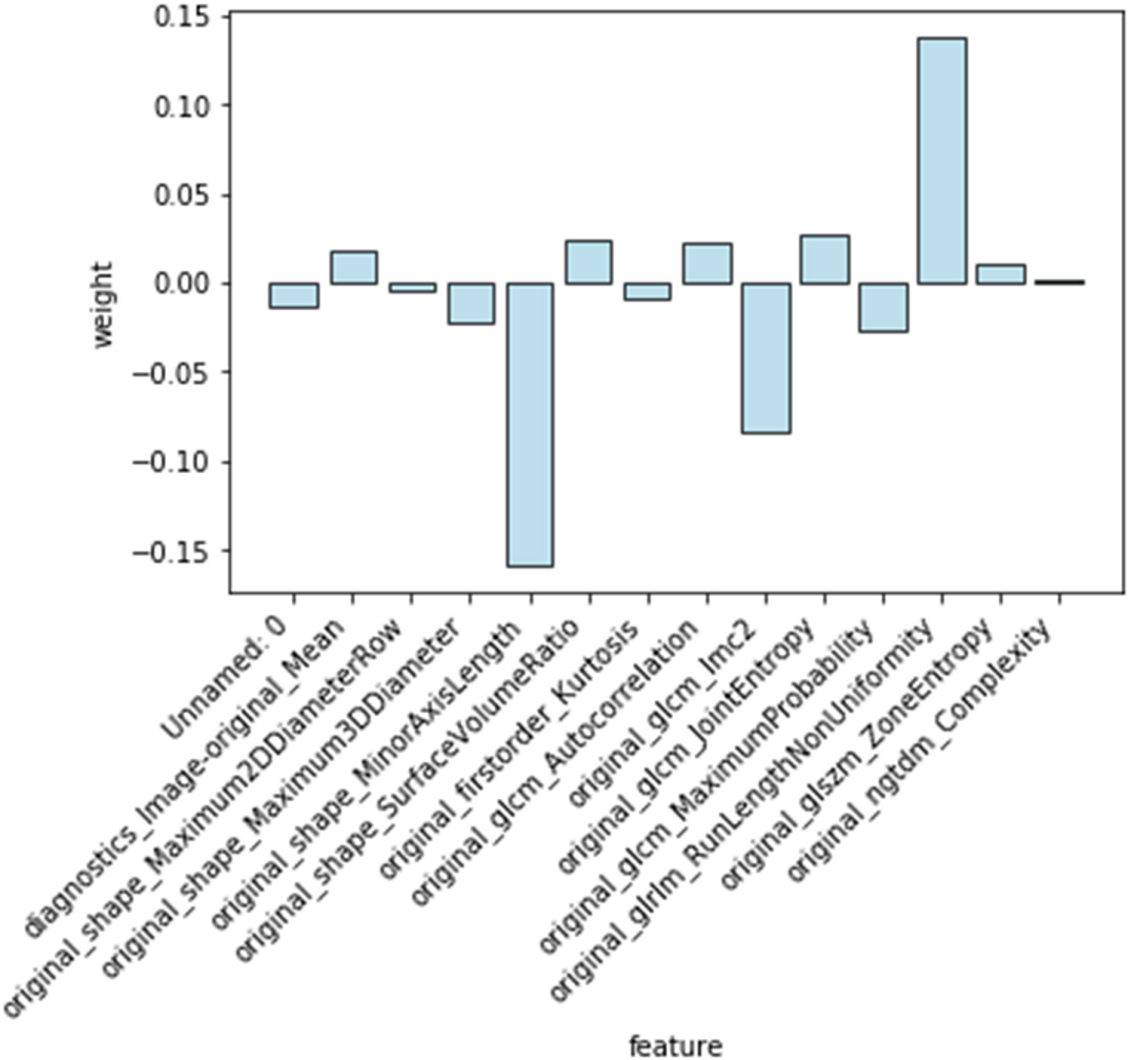
Selected features and weights. Fourteen features were extracted, which were showed on *X*-axis. The weights of features were displayed on *Y*-axis. The positive or negative weights indicate positive or negative correlation with AF.

The Radiomics score was calculated by the feature values of the selected 12 feature variables multiplied with their corresponding weights, and then added together. Steps: (1) The feature values were obtained as followings: after drawing the regions of interest (ROI) on myocardium of each patient, Pyradiomics feature extractor was used to extract features, and the values of features were showing simultaneously. In the formula, the values of Feature *n*(*n* = 1,2,3..,12) is varied from person to person. (2) 14 features were selected with their weights by LASSO. Because of the data redundant, only 12 features remained. These values of 12 features were multiplied with their corresponding weights, which were also called coefficients (shown in Figure 8). (3) They were added together to calculate the radiomics score.

It was found that the Radiomics score of the AF group was lower than that of the control group. The predictive power of Radiomics score for reflecting radiomics model is 0.825.

Radiomics score formula:

Rad-score = −0.005*feature1- 0.022*feature2 −0.158*feature3 + 0.024*feature4 −0.010*feature5 + 0.023*feature6 −0.083*feature7 + 0.027*feature8 −0.027*feature9 + 0.138*feature10 + 0.011*feature11 + 0.001*feature12

### 5.3 Establishment of a model based on a combination of radiomics and clinical features

**Figure 9:**
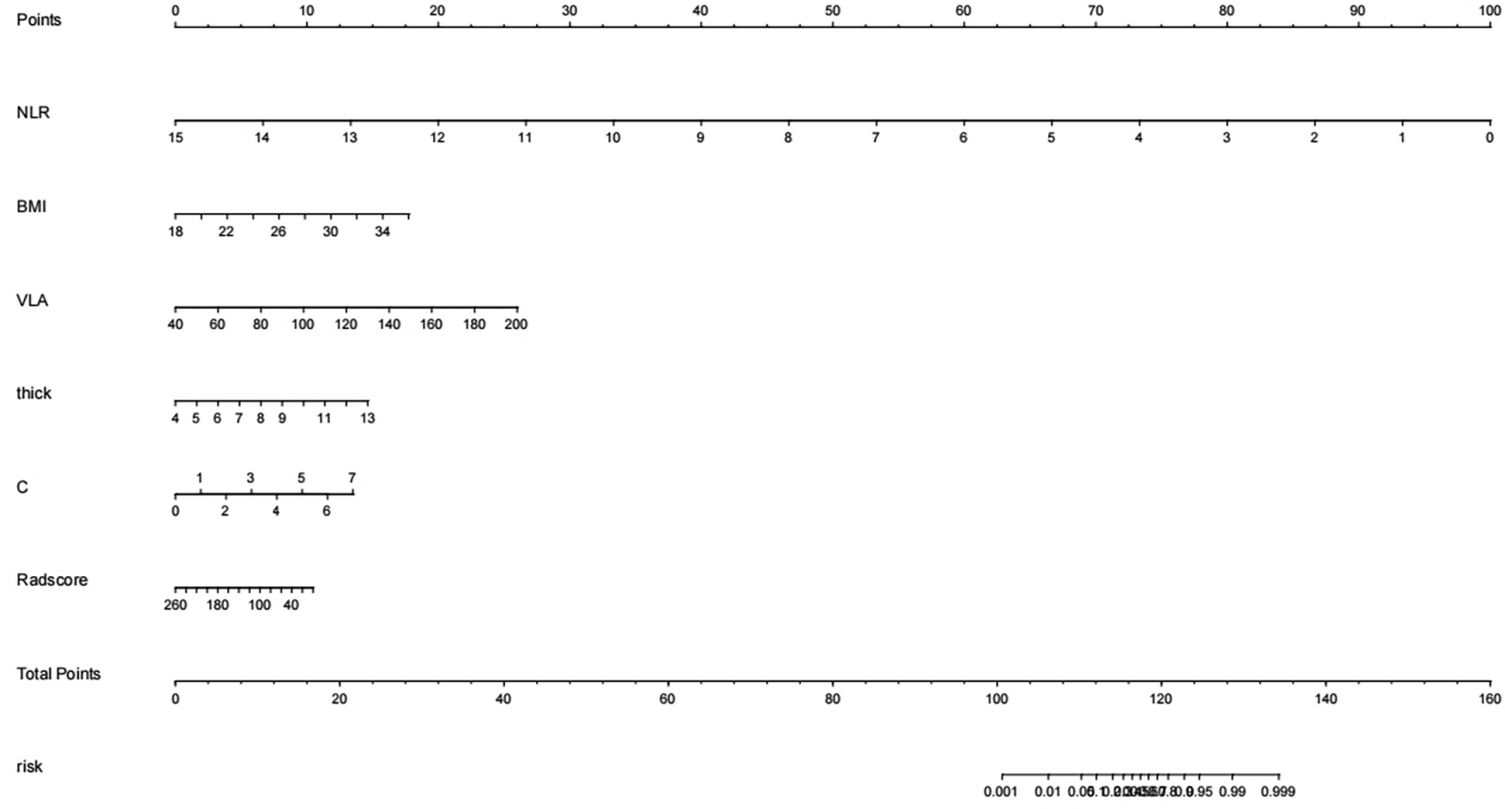
The nomogram of a predictive model combining radiomics and clinical features.

A predictive model for AF was established by combining BMI, LA volume, the myocardial thickness at the junction of LAA and LA, NLR, CHA2DS-VASC score, and radiomics score. The model was ultimately presented in a nomogram (Figure 9). The nomogram establishes variable scales based on the weights of the regression coefficients for all independent variables. It connects each known variable through contour lines, assigns corresponding scores to each independent variable, and calculates a total score by summing up the scores of each variable. The patient's total score is then located on the total score axis. Drawing a line perpendicular to the risk probabilit*y* axis from this point provides the estimated risk of the patient experiencing the event. This study will use multivariable logistic stepwise regression analysis to construct a nomogram based on radiomic features and clinical risk factors, which visually displays the range of variable values and their contributions to the risk, making the results of the prediction model more interpretable.

### 5.4 Comparison of predictive models

**Figure 10:**
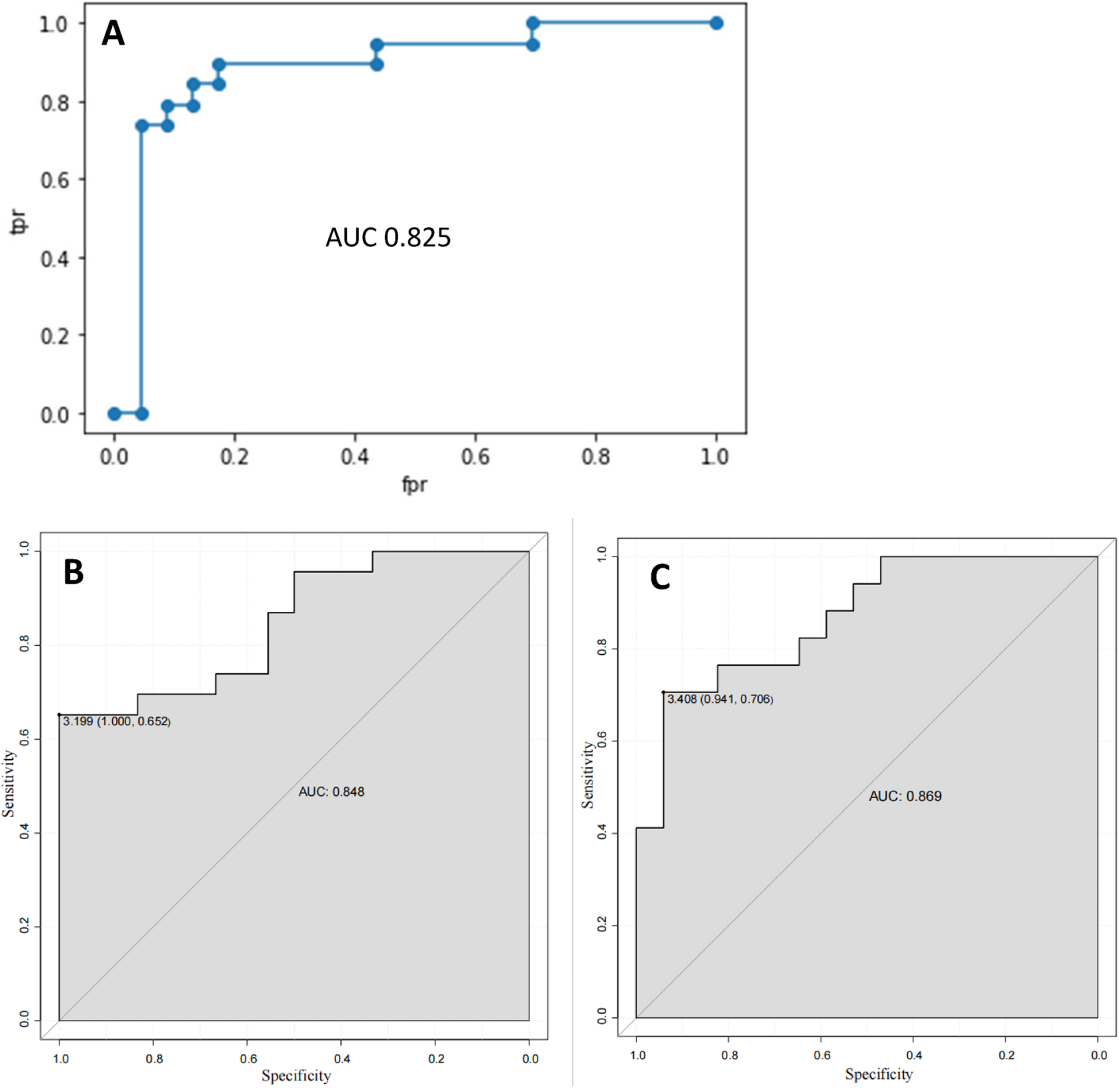
The ROC curve. **(A)** The ROC curve of the radiomics model; **(B)** The ROC curve of the clinical feature model; **(C)** The ROC curve of the model combining radiomics and clinical features.

The radiomics model, the clinical feature model and the combined model were compared, respectively. The combined model, which integrates radiomics with clinical features, achieved a higher AUC (0.869) compared to the radiomics model (AUC = 0.825) and the clinical features model (AUC = 0.848). ROC curves for the radiomics model, the clinical features model and the combined model were obtained (Figure 10). The accuracy, specificity and sensitivity of the combined model, which integrates radiomics with clinical features model are 0.88, 0.82, 0.92 (Table 8).

**Table 8:** Accuracy, specificity and sensitivity of the three models.

	Radiomics model		Clincal feature model		Model combining radiomics and clinical feature	
	Training	Testing	Training	Testing	Training	Testing
Accuracy	0.91	0.83	0.84	0.85	0.87	0.88
Specificity	0.93	0.91	0.87	0.76	0.92	0.82
Sensitivity	0.86	0.74	0.81	0.91	0.83	0.92

### 5.5 Calibration curve

**Figure 11:**
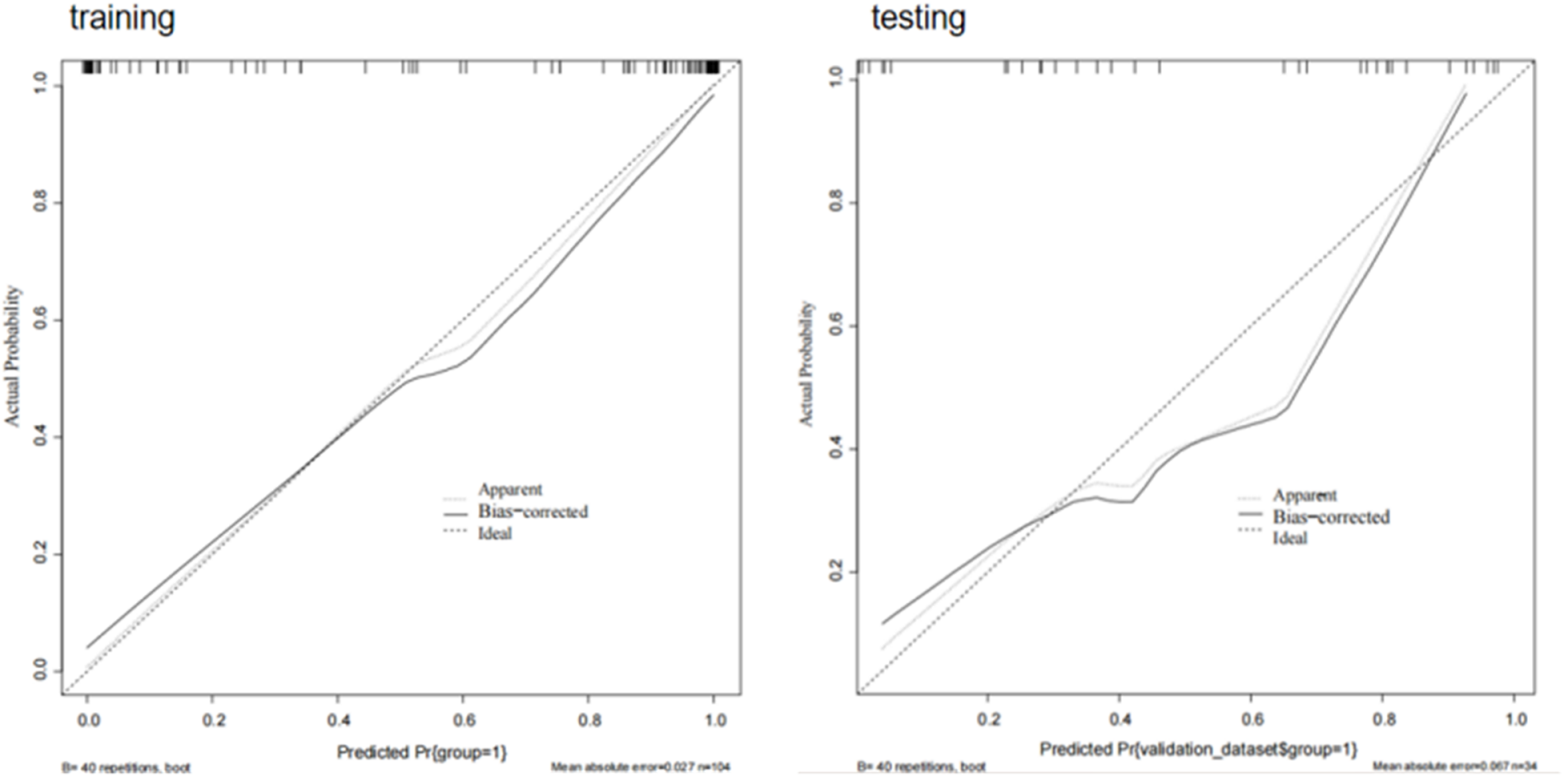
Calibration curves of combining radiomics and clinical features. When the solid line (predicted situation) is closer to the dashed line (actual situation), the calibration effect of the model is better.

Calibration curves for the model based on a combination of radiomics and clinical features showed good fit between prediction and observation of AF in the training and test sets (Figure 11).

### 5.6 Decision curve

**Figure 12:**
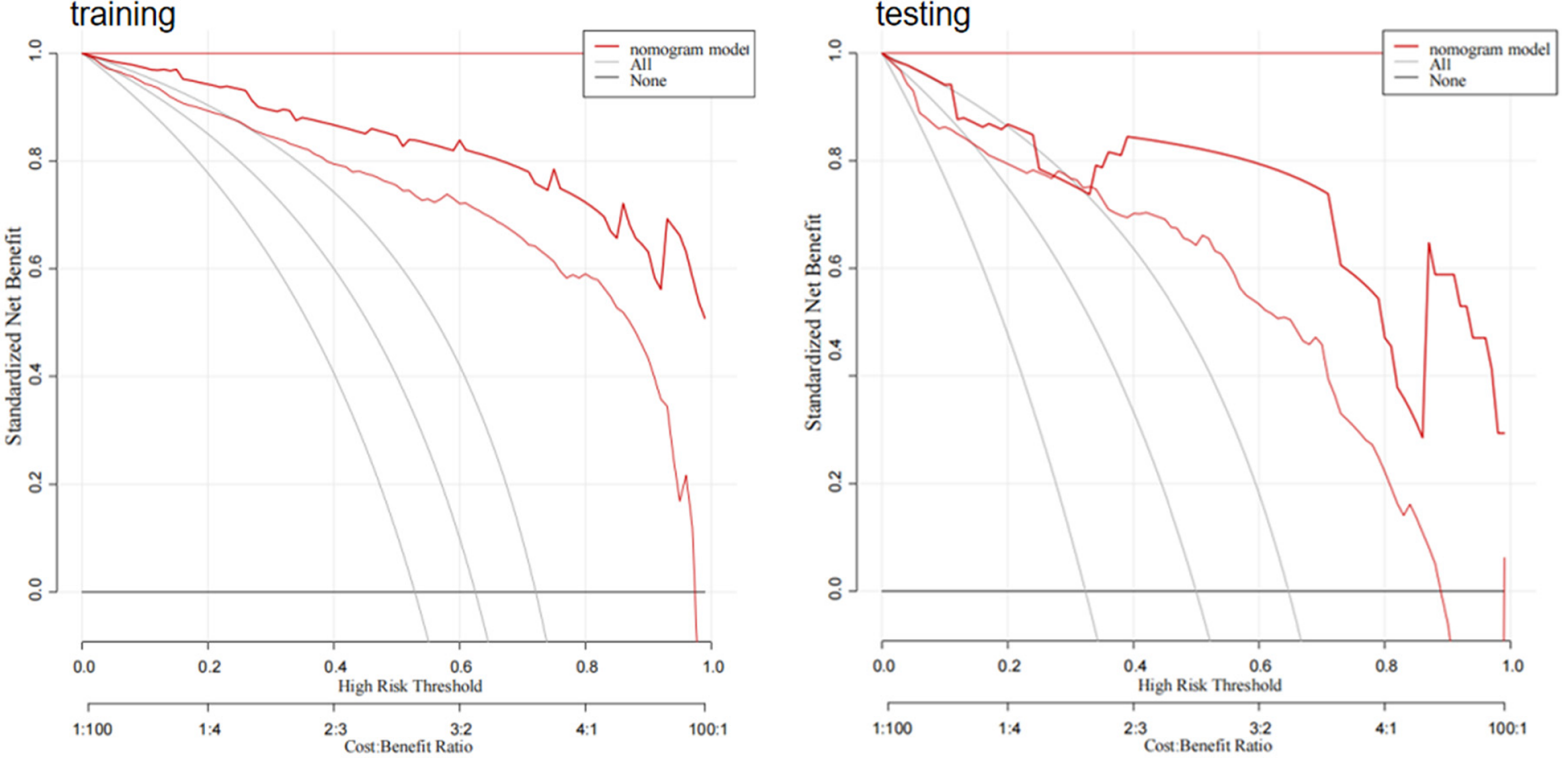
Decision curve. The *Y*-axis represents the net benefit and the *X*-axis represents the threshold probability. The gray curve represents the hypothesis that all patients develop AF events, and the black curve represents the hypothesis that no patients develop AF.

The decision curve demonstrated the clinical utility of the model based on a combination of radiomics and clinical features by comparing the net benefits across various threshold probabilities in both the training and testing sets. The curve indicated that the model yielded substantial net benefits, representing its effectiveness (Figure 12).

## 6 Discussion

This article investigated radiomic features extracted from the myocardium at the junction of the LAA and LA to establish predictive models. Three models were developed: a radiomics model of the myocardium at the junction of the LAA and LA, a clincal feature model and a combined model incorporating both radiomic and clinical features. The combined model superiorly predicted the risk of AF. This represents a novel endeavor, monitoring AF by observing the myocardium. Multivariate analysis was conducted to exclude confounding factors, revealing that BMI, myocardial thickness at the junction of the LAA and LA, LA volume, NLR, and CHA2DS-VASC score were independent risk factors for AF. In contrast, age, hypertension, LAA circumference, LAA ostium area, and LAA ostium long/short diameter showed no association with AF, consistent with previous studies (27). We found that although LAA volume related with AF, it is not an independent factor for AF. The reason might be that hypertension patients were enrolled in both groups, and hypertension caused increasing of the volume of the LA and LAA. Some studies have shown that the LAA is more sensitive to hypertension (28). NLR is associated with AF, which is consistent with the Lu M study (29). Furthermore, the CHA2DS-VASC score is associated with stroke caused by AF (24).

Researches have indicated that AF patients commonly exhibit extensive fibrosis in the myocardium of the LAA, accompanied by myocardial fiber degeneration, elongation of myocardial cell sarcomeres, increased intercellular matrix, and collagen production, leading to a reduction in LAA and LA emptying fraction (30). These alterations are believed to contribute to radiomic characteristics (31). Our study leveraged radiomics to extract myocardial characteristics and to evaluate subtle changes within the myocardium. In the formula of Rad-score, the larger coefficients were associated with the feature 10 and feature 3. The feature 10 was the Run Length Non Uniformity, which indicated potential fibrosis of the myocardium. The feature 3 was the Minor Axial Length, which indicated the shape of myocardium at the junction of the LAA and LA changed in AF patients. Therefore, radiomic model can identify fibrosis and morphological change of myocardium. The AUC of the radiomic feature model to predict AF was 0.825. Rad-score is a comprehensive index of radiomics features, which combines multiple radiomics features into one value to predict AF. The lower of the Radcore, the higher the risk of AF.

The nomogram showed that the thicker the myocardium and the larger volume of the LA, the lower the radiomics score, the higher the risk of AF. ROC curve analysis confirmed the model, based on both radiomics features and clinical factors, can effectively predict AF. The Calibration curves and the decision curve indicated that the model exhibited a good predictive capability.

We found that there is a correlation between LA volume and the myocardial thickness at the junction of LAA and LA, while there is no correlation between the onset time of AF and myocardial thickness. When evaluating myocardial changes in AF patients, we may need to consider changes in LA volume rather than the onset time of AF.

The reproducibility and robustness are important for a imaging research. To ensure the reproducibility, we followed standardized protocols and methodologies as mentioned in “Methods” section. Pre-training and discussion between observers when they had different opinions are helpful to obtain consistent results. To ensure the robustness, we enrolled patients with high quality images with same CT protocols.

## 7 Limitations

This study only included cases from one center, lacking multi center and large sample size for dataset training and validation. Nonetheless, the study underscores the potential predictive value of CT radiomics, particularly based on myocardial thickness at the junction of LAA and LA, in assessing the risk of AF.

## 8 Conclusions

We have demonstrated the feasibility of using myocardial radiomics features at the junction of LAA and LA to predict the risk of AF. The Rad score of the AF group was lower than that of the control group. The lower the Rad score, the higher the risk of AF. The combination of clinical features and myocardial radiomics features has superior predictive capability of AF, providing reliable evidence that radiomic features of the myocardium at the junction of the LAA and LA might predict AF.

## Data Availability

The raw data supporting the conclusions of this article will be made available by the authors, without undue reservation.

## References

[1] Malladi V Naeini PS Razavi M Collard CD Anton JM Tolpin DA. Endovascular ablation of atrial fibrillation. Anesthesiology. (2014) 120(6):1513–9. 10.1097/ALN.0000000000000261 24714120

[2] Andersen JH Andreasen L Olesen MS. Atrial fibrillation-a complex polygenetic disease. Eur J Hum Genet. (2021) 29(7):1051–60. 10.1038/s41431-020-00784-8 33279945 PMC8298566

[3] Chen L Xu C Chen W Zhang C. Left atrial appendage orifice area and morphology is closely associated with flow velocity in patients with nonvalvular atrial fibrillation. BMC Cardiovasc Disord. (2021) 21(1):442. 10.1186/s12872-021-02242-9 34530731 PMC8443967

[4] Takaya Y Nakayama R Yokohama F Toh N Nakagawa K Miyamoto M Left atrial appendage morphology with the progression of atrial fibrillation. PLoS One. (2022) 17(11):e0278172. 10.1371/journal.pone.0278172 36449497 PMC9710751

[5] Yosefy C Pery M Nevzorov R Piltz X Osherov A Jafari J Difference in left atrial appendage remodeling between diabetic and nondiabetic patients with atrial fibrillation. Clin Cardiol. (2020) 43(1):71–7. 10.1002/clc.23292 31755572 PMC6954381

[6] Słodowska K Hołda J Dudkiewicz D Malinowska K Bolechała F Kopacz P Thickness of the left atrial wall surrounding the left atrial appendage orifice. J Cardiovasc Electrophysiol. (2021) 32(8):2262–8. 10.1111/jce.15157 34245483

[7] Sheng Y Wang YY Chang Y Ye D Wu L Kang H Deciphering mechanisms of cardiomyocytes and non-cardiomyocyte transformation in myocardial remodeling of permanent atrial fibrillation. J Adv Res. (2024) 61:101–17. 10.1016/j.jare.2023.09.012 37722560 PMC11258668

[8] Ma S Ma J Tu Q Zheng C Chen Q Lv W. Isoproterenol increases left atrial fibrosis and susceptibility to atrial fibrillation by inducing atrial ischemic infarction in rats. Front Pharmacol. (2020) 11:493. 10.3389/fphar.2020.00493 32351393 PMC7174760

[9] Lambin P Rios-Velazquez E Leijenaar R Carvalho S van Stiphout RG Granton P Radiomics: extracting more information from medical images using advanced feature analysis. Eur J Cancer. (2012) 48(4):441–6. 10.1016/j.ejca.2011.11.036 22257792 PMC4533986

[10] Kumar V Gu Y Basu S Berglund A Eschrich SA Schabath MB Radiomics: the process and the challenges. Magn Reson Imaging. (2012) 30(9):1234–48. 10.1016/j.mri.2012.06.010 22898692 PMC3563280

[11] Li G Li L Li Y Qian Z Wu F He Y An MRI radiomics approach to predict survival and tumour-infiltrating macrophages in gliomas. Brain. (2022) 145(3):1151–61. 10.1093/brain/awab340 35136934 PMC9050568

[12] Binczyk F Prazuch W Bozek P Polanska J. Radiomics and artificial intelligence in lung cancer screening. Transl Lung Cancer Res. (2021) 10(2):1186–99. 10.21037/tlcr-20-708 33718055 PMC7947422

[13] Balagurunathan Y Gu Y Wang H Kumar V Grove O Hawkins S Reproducibility and prognosis of quantitative features extracted from CT images. Transl Oncol. (2014) 7(1):72–87. 10.1593/tlo.13844 24772210 PMC3998690

[14] Wu Y Xu L Yang P Lin N Huang X Pan W Survival prediction in high-grade osteosarcoma using radiomics of diagnostic computed tomography. EBioMedicine. (2018) 34:27–34. 10.1016/j.ebiom.2018.07.006 30026116 PMC6116348

[15] Zhang YF Zhou C Guo S Wang C Yang J Yang ZJ Deep learning algorithm-based multimodal MRI radiomics and pathomics data improve prediction of bone metastases in primary prostate cancer. J Cancer Res Clin Oncol. (2024) 150(2):78. 10.1007/s00432-023-05574-5 38316655 PMC10844393

[16] Dai P Chang W Xin Z Cheng H Ouyang W Luo A. Retrospective study on the influencing factors and prediction of hospitalization expenses for chronic renal failure in China based on random forest and LASSO regression. Front Public Health. (2021) 9:678276. 10.3389/fpubh.2021.678276 34211956 PMC8239170

[17] Wu W Parmar C Grossmann P Quackenbush J Lambin P Bussink J Exploratory study to identify radiomics classifiers for lung cancer histology. Front Oncol. (2016) 6:71. 10.3389/fonc.2016.00071 27064691 PMC4811956

[18] Yang M Cao Q Xu Z Ge Y Li S Yan F Development and validation of a machine learning-based radiomics model on cardiac computed tomography of epicardial adipose tissue in predicting characteristics and recurrence of atrial fibrillation. Front Cardiovasc Med. (2022) 9:813085. 10.3389/fcvm.2022.813085 35310976 PMC8927627

[19] Pujadas ER Raisi-Estabragh Z Szabo L Morcillo CI Campello VM Martin-Isla C Atrial fibrillation prediction by combining ECG markers and CMR radiomics. Sci Rep. (2022) 12(1):18876. 10.1038/s41598-022-21663-w 36344532 PMC9640662

[20] Firouznia M Feeny AK LaBarbera MA McHale M Cantlay C Kalfas N Machine learning-derived fractal features of shape and texture of the left atrium and pulmonary veins from cardiac computed tomography scans are associated with risk of recurrence of atrial fibrillation postablation. Circ Arrhythm Electrophysiol. (2021) 14(3):e009265. 10.1161/CIRCEP.120.009265 33576688 PMC8015207

[21] Niemann B Doll N Grubitzsch H Hanke T Knaut M Senges J Surgical ablation of atrial fibrillation in high-risk patients: success versus risk. Thorac Cardiovasc Surg. (2024). 10.1055/a-2334-9039 38806162

[22] Visseren FLJ Mach F Smulders YM Carballo D Koskinas KC Bäck M 2021 ESC guidelines on cardiovascular disease prevention in clinical practice. Eur Heart J. (2021) 42(34):3227–337. 10.1093/eurheartj/ehab484 34458905

[23] Hołda MK Hołda J Strona M Koziej M Klimek-Piotrowska W. Blood vessels and myocardial thickness within the left atrial appendage isthmus line. Clin Anat. (2018) 31(7):1024–30. 10.1002/ca.23242 30069992

[24] Beinart R Heist EK Newell JB Holmvang G Ruskin JN Mansour M. Left atrial appendage dimensions predict the risk of stroke/TIA in patients with atrial fibrillation. J Cardiovasc Electrophysiol. (2011) 22(1):10–5. 10.1111/j.1540-8167.2010.01854.x 20662984

[25] Zheng YM Chen J Xu Q Zhao WH Wang XF Yuan MG Development and validation of an MRI-based radiomics nomogram for distinguishing Warthin's tumour from pleomorphic adenomas of the parotid gland. Dentomaxillofac Radiol. (2021) 50(7):20210023. 10.1259/dmfr.20210023 33950705 PMC8474129

[26] Ye Z Zhu Y Coffman DL. Variable selection for causal mediation analysis using LASSO-based methods. Stat Methods Med Res. (2021) 30(6):1413–27. 10.1177/0962280221997505 33755518 PMC8189011

[27] Tian X Wang C Gao D Gao BL Li CY. Morphological changes in the orifices of the left atrial appendage and left atrium in patients with atrial fibrillation. Quant Imaging Med Surg. (2022) 12(12):5371–82. 10.21037/qims-22-218 36465818 PMC9703112

[28] Lakkireddy D Turagam M Afzal MR Rajasingh J Atkins D Dawn B Left atrial appendage closure and systemic homeostasis. J Am Coll Cardiol. (2018) 71(2):135–44. 10.1016/j.jacc.2017.10.092 29325636

[29] Lu M Zhang Y Liu R He X Hou B. Predictive value of neutrophil to lymphocyte ratio for ischemic stroke in patients with atrial fibrillation: a meta-analysis. Front Neurol. (2022) 13:1029010. 10.3389/fneur.2022.1029010 36578303 PMC9792176

[30] McMillan AB Shi D Pratt SJP Lovering RM. Diffusion tensor MRI to assess damage in healthy and dystrophic skeletal muscle after lengthening contractions. J Biomed Biotechnol. (2011) 2011:970726. 10.1155/2011/970726 22190860 PMC3228693

[31] Kolossváry M Kellermayer M Merkely B Maurovich-Horvat P. Cardiac computed tomography radiomics: a comprehensive review on radiomic techniques. J Thorac Imaging. (2018) 33(1):26–34. 10.1097/RTI.0000000000000268 28346329

